# CD1a^+^ survivin^+^ dendritic cell infiltration in dermal lesions of systemic sclerosis

**DOI:** 10.1186/s13075-015-0785-0

**Published:** 2015-09-30

**Authors:** Sho Mokuda, Tatsuhiko Miyazaki, Yoshifumi Ubara, Masamoto Kanno, Eiji Sugiyama, Kiyoshi Takasugi, Junya Masumoto

**Affiliations:** Department of Immunology, Graduate School of Biomedical and Health Sciences, Hiroshima University, 1-2-3 Kasumi, Minami-ku, Hiroshima 734-8551 Japan; Department of Pathology, Ehime University Proteo-Science Centre and Graduate School of Medicine, Shizukawa, Toon, Ehime 791-0295 Japan; Department of Internal Medicine, Center for Rheumatic Diseases, Dohgo Spa Hospital, 21-21 Otsu Dohgo-Himezuka, Matsuyama, Ehime 790-0858 Japan; Department of Clinical Immunology and Rheumatology, Hiroshima University Hospital, 1-2-3 Kasumi, Minami-ku, Hiroshima 734-8551 Japan; Nephrology Center and the Okinaka Memorial Institute for Medical Research, Toranomon Hospital, 2-2-2 Toranomon, Minato-ku, Tokyo 105-8470 Japan

## Abstract

**Introduction:**

Proto-oncogene survivin is a member of the inhibitor of apoptosis (IAP) family of proteins. The presence of serous antibodies against survivin in patients with systemic sclerosis has been previously reported; however, there are few reports regarding the pathophysiological relationship between survivin and systemic sclerosis. We herein investigated the expression and function of survivin in SSc patients.

**Methods:**

We performed immunohistochemistry analyses to determine the expression of XIAP, cIAP and survivin in skin lesions from patients with SSc and non-SSc. The expression levels of survivin in peripheral blood mononuclear cells (PBMCs) obtained from SSc patients and healthy controls were evaluated using RT-PCR and flow cytometry. Additionally, the function of survivin was verified with overexpression experiments using monocyte-derived dendritic cells (Mo-DCs).

**Results:**

The expression patterns of both XIAP and cIAP were similar, while only the survivin expression differed between the SSc and non-SSc skin lesions. Survivin-overexpressing cells were detected in the SSc dermis frequently. The positive rate of survivin in SSc dermis (64.3 %, 9/14) was higher than that in non-SSc dermis (11.2 %, 1/9). Furthermore, survivin^+^ cells expressed CD1a, one of the DC markers. Real-time PCR and FACS analyses revealed that the survivin-WT (wild type) expression levels in PBMCs, in particular CD14^+^ monocytes, from SSc patients were higher than that from healthy controls. Additionally, the overexpression experiments showed that survivin-WT-overexpressing CD1a^+^ Mo-DCs have the characteristics of promoting cell cycle progression and decreasing apoptotic cells.

**Conclusions:**

These findings suggest that dermal survivin^+^ CD1a^+^ cell infiltration may be a potential biomarker of SSc skin lesions. PBMCs and monocytes from SSc patients also overexpressed survivin; therefore, dermal survivin^+^ DC may be derived from peripheral blood monocytes. Additionally, survivin may be involved in dermal CD1a^+^ DC proliferation through cell cycle activation and resistance to apoptosis. Survivin may be an important molecule for the pathogenesis of SSc.

## Introduction

Survivin is a member of the inhibitor of apoptosis (IAP) family of proteins, which is characterized by several baculovirus IAP repeat (BIR) domains [1, 2]. It is encoded by the baculoviral IAP repeat containing 5 (*BIRC5*) gene on human chromosome 17q25, which encodes a 16.5 kDa protein of 142 amino acids. Survivin plays pivotal roles in many fundamental cellular processes, including cell division and apoptosis [3], and has been suggested to be involved in cancer development, progression and resistance to treatment [4]. Survivin is especially expressed during normal embryonic development and in human cancers, while most normal differentiated cells do not express survivin [5]; therefore, survivin has attracted attention as a potential target for cancer therapy [6].

Recently, it has been demonstrated that some autoimmune diseases (for example, rheumatoid arthritis and multiple sclerosis) are associated with IAP family proteins [7, 8]. Systemic sclerosis (SSc) is an autoimmune disease characterized by fibrosis of the skin and other organs, obliterative vasculopathy and immunological abnormalities. In 2010, it has been reported that autoantibody against survivin was detectable in patients with SSc [9]. However, the pathophysiological relationship between SSc and IAPs, including survivin, remains unclear. In the present study, we investigated the expression of IAPs in SSc patients and tried to show the function of survivin in SSc patients.

## Methods

### Patients

This study was conducted at the Dohgo Spa Hospital, Toranomon Hospital, and Ehime University Graduate School of Medicine, and was approved by the clinical ethics committees of these institutions. SSc, rheumatoid arthritis (RA), polymyositis/dermatomyositis (PM/DM), osteoarthritis (OA) patients and healthy volunteers, who were provided adequate information and signed a written consent form, were included in this study. Patients who suffered from cutaneous cancer, hematologic cancer and any cutaneous infection were excluded. Clinical data were obtained from medical records. All SSc patients fulfilled the preliminary American College of Rheumatology (ACR) criteria for classification in 1980 [10]. Patients with SSc were divided into those with limited and diffuse diseases according to a previous report [11]. Interstitial pneumonia (IP) based on SSc was defined as the change in the bilateral basilar lung portions, which were detected with high-resolution computed tomography [12]. The definition of scleroderma renal crisis (RC) was according to a previous report in 2007 [13]. The existence of pulmonary artery hypertension (PAH) was demonstrated with echocardiography, and subsequent right heart catheterization was performed to confirm the diagnosis [14].

### Immunohistochemistry (IHC)

The skin specimens of SSc were collected from the forearms, and those of the control patients were collected from the forearms or lower legs. Formalin-fixed paraffin-embedded tissue sections were used for the IHC studies. The following primary antibodies were employed: anti-XIAP (rabbit polyclonal, Abcam, Tokyo, Japan), anti-cIAP (cIAP1 and cIAP2) (mouse monoclonal, clone number 681724, R&D Systems, Minneapolis, MN, USA), anti-survivin (rabbit polyclonal, R&D Systems), anti-survivin-ΔEx3 (rabbit polyclonal, Abcam), anti-survivin-2B (rabbit polyclonal, Abcam), anti-CD1a (mouse monoclonal, clone 7A7, Abcam), anti-CD4 (mouse monoclonal, clone CRRY77, eBioscience, Affymetrix Japan K.K., Tokyo, Japan) and anti-CD69 (rabbit polyclonal, Bioss, Woburn, MA, USA). Following deparaffinization and antigen retrieval in citrate buffer (pH 6.0), the specimens were incubated with primary antibodies followed by horseradish peroxidase (HRP)-conjugated secondary antibodies and then visualized with the 3-amino-9-ethylcarbazole (AEC) + kit (Agilent Technologies Dako, Tokyo, Japan). Multiple immunofluorescence staining was also conducted using an indirect method with Alexa Fluor-conjugated secondary antibodies (Invitrogen, Thermo Fisher Scientific, Yokohama, Japan). The slides were also stained with hematoxylin, Hoechst 33342 or 4′,6-diamidino-2-phenylindole, dihydrochloride (DAPI). The fluorescence was captured with an Olympus FV1000D laser scanning microscope (Olympus, Tokyo, Japan) and a Leica 6000E microscope (Leica microsystems, Tokyo, Japan).

### Enzyme-linked immunosorbent assay (ELISA)

Three types of SSc-specific autoantibodies (anti-topoisomerase I (Scl-70), anti-centromere and anti-RNA polymerase III (RNAPIII)) were measured with MESACUP ELISA kits (Medical & Biological Laboratories, Nagoya, Japan). For the detection of serum anti-survivin immunoglobulin G (IgG) antibody, an ELISA was performed as previously described [9]. Briefly, 96-well plates were coated with glutathione S-transferase (GST) fusion recombinant proteins of survivin-wild type (WT) (1,000 ng/ml), which were prepared as previously reported [15]. The wells were blocked with Blocking One P (Nacalai Tesque, Kyoto, Japan). Serum samples were diluted 1:100 in Tris-buffered saline (TBS) containing Blocking One P and added to the wells. After incubation and washing, alkaline phosphatase-conjugated goat anti-human IgG antibody was added. Following additional washing, p-nitrophenyl phosphate (Pierce, Thermo Fisher Scientific, Waltham, MA, USA) was added and measured at 405 nm on a spectrophometer. The mean + 2SD (standard deviation) among the healthy controls (HCs) (n = 15, data not shown) was defined as the cutoff value for anti-survivin IgG antibody.

### Reverse transcription polymerase chain reaction (RT-PCR) and real-time PCR

Peripheral blood mononuclear cells (PBMCs) from SSc patients and HCs were enriched using Ficoll-Paque PLUS (GE Healthcare, Tokyo, Japan). Total RNA of the PBMCs or cultured cells was extracted and purified using TRIzol (Invitrogen) followed by cDNA synthesis using a PrimeScript RT Reagent Kit with a gDNA Eraser (Takara-Bio, Shiga, Japan). Quantitative real-time PCR using TaqMan probes (FAM/TAMRA) and SYBR Green was executed in triplicate using the ABI Prism 7500 Real-Time PCR system (Applied Biosystems, Thermo Fisher Scientific, Yokohama, Japan). The HL-60 cell line was used as a positive control for RT-PCR. The primer pairs and probes for amplification of human BIRC5 variants and hypoxanthine-guanine phosphoribosyltransferase (HPRT) were described previously [15]. The upstream and downstream primer sequences used for interleukin (IL)-6 were 5′-GAGAAAGGAGACATGTAACAAGAGT-3′ and 5′-GCAAGTCTCCTCATTGAATCCA-3′, respectively. The upstream and downstream primer sequences for IL-12B were 5′-CCAGAGCAAGATGTGTCAC-3′ and 5′-CTACGACATAAACATCTTTCTTCAG-3′, respectively. The expression of human HPRT was evaluated as a housekeeping gene to normalize the ΔCt values. The relative expressions of the target genes were obtained using the difference in the comparative threshold (ΔΔCt) method.

### Fluorescence-activated cell sorting (FACS)

A flow cytometric analysis was performed using the FACSCalibur and LSRFortessa platforms (BD Biosciences, Tokyo, Japan). For Ki-67 and 7-amino-actinomycinD (7-AAD) staining, the FOXP3 Staining Buffer Set (eBioscience) was used. Antibodies conjugated with FITC, PerCP or APC were used. Anti-human CD14 (clone MφP9), anti-human CD45 (clone 2D1) antibodies and 7-AAD were purchased from BD Biosciences. Anti-human CD1a (clone HI149), anti-human Ki-67 (clone Ki-67) antibodies and Fixable Viability Dye (FVD520) were obtained from eBioscience. The cells were washed and stained. For intracellular staining of survivin and its splice variants, normal rabbit IgG (Santa Cruz Biotechnology, TX, USA), anti-survivin (rabbit polyclonal, R&D Systems), anti-survivin-ΔEx3 (rabbit polyclonal, Abcam), and anti-survivin-2B (rabbit polyclonal, Abcam) were labeled using the Zenon Rabbit IgG Labeling Kit (Alexa Fluor 647, Invitrogen). The FOXP3 Staining Buffer Set (eBioscience) was used for fixation and permeabilization. A survivin-WT-specific antibody was not available, therefore, the mean fluorescence intensity (MFI) of the estimated survivin-WT expression level was calculated from other MFI parameters as follows: (survivin-WT) = (survivin (total) – normal IgG) – (survivin-ΔEx3 – normal IgG) – (survivin–2B – normal IgG).

### Preparation of monocyte-derived dendritic cells (Mo-DCs)

Human monocytes were purified with biotin-conjugated anti-CD14 antibody (clone 61D3, eBioscience) and streptavidin microbeads (Miltenyi Biotec, Tokyo, Japan) from PBMCs, which were enriched using Ficoll-Paque PLUS (GE Healthcare). CD14^+^ monocytes isolation resulted in >95 % pure population. Mo-DCs were generated by culturing CD14^+^ monocytes in RPMI1640 medium supplemented with 10 % fetal bovine serum (Gibco, Thermo Fisher Scientific, Yokohama, Japan), 200 ng/ml rhIL-4 (R&D Systems) and 200 ng/ml rhGM-CSF (Wako Junyaku Kogyo, Tokyo, Japan). After 5 days of culture, CD1a^+^CD14^−^ Mo-DCs (>95 % CD1a^+^ purity) were harvested for use.

### Transfection for dendritic cells (DCs)

The transfection of in vitro transcribed (IVT) messenger RNA (mRNA) for DCs was performed using Lipofectamine MessengerMAX Transfection Reagent (Invitrogen). To generate templates for IVT mRNA, we developed the pcDNA3-A(124) vector plasmid and *BIRC5*-inserted vector plasmids from artificial custom genes, according to a protocol described previously [16, 17]. The transcription of pcDNA3-A(124)-based plasmids was performed with the mMESSAGE mMACHINE T7 transcription Kit (Ambion, Thermo Fisher Scientific, Yokohama, Japan). After in vitro transcription, mRNA was purified using the MEGAclear Kit (Ambion).

### Western blot

Cell lysates and recombinant proteins were processed using SDS-PAGE and Western blotting according to the standard procedures. The blotted membranes were incubated with anti-actin (mouse monoclonal, clone AC-15, Invitrogen) and anti-survivin antibodies followed by HRP-conjugated secondary antibodies (Jackson ImmunoResearch Inc., West Grove, PA, USA). The GST-fusion recombinant proteins of survivin variants were prepared as previously reported [15].

### Statistical analysis

The significance in the differences between the two groups was determined using an unpaired *t* test, the Mann–Whitney *U* test or the chi-square test. The data processing and analyses were conducted using the Microsoft Excel software program (Microsoft, Tokyo, Japan).

## Results

### The expression of IAPs and survivin in SSc skin lesions detected with IHC

**Fig. 1:**
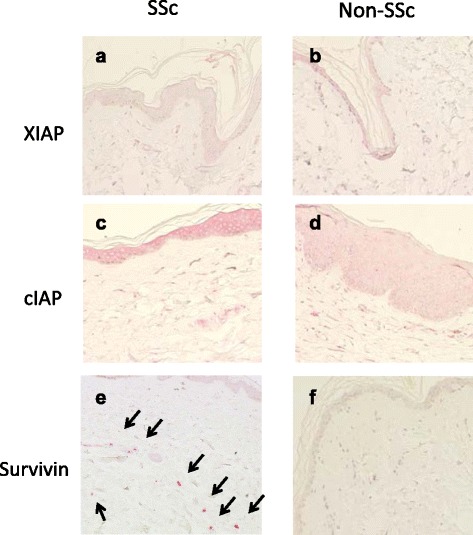
Immunohistochemistry (IHC) staining of XIAP, cIAP and survivin in systemic sclerosis (SSc) dermal lesions. SSc (**a**, **c**, **e**) and non-SSc (**b**, **d**, **f**) skin lesions evaluated with IHC (all stained by red, ×20). The SSc patients were designated numbers *4*, *6*, *7*, and *12* in Table 1 (n = 4). A case of rheumatoid arthritis (RA), a case of polymyositis/dermatomyositis (PM/DM) and two cases of osteoarthritis (OA) were included among the non-SSc patients (n = 4). **a**, **b** XIAP. **c, d** cIAP. **e**, **f** survivin. Representative images are shown. In the dermis of SSc, many survivin-positive cells were detected (*arrows*)

**Fig. 2:**
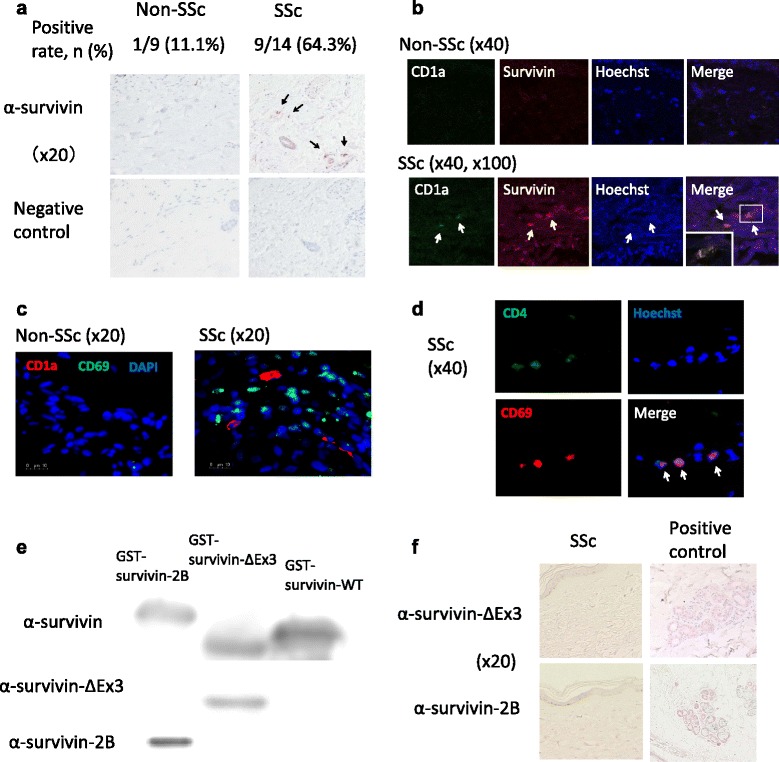
The characteristics of survivin expression in systemic sclerosis (SSc) dermal lesions. **a** *Arrows* indicate survivin-positive cells (stained by red). (**b**-**d**) The multiple immunofluorescence method for SSc and non-SSc dermal lesions with anti-survivin, anti-CD1a, anti-CD4 and anti-CD69 antibodies was performed. **b** *Arrows* indicate CD1a^+^ survivin^+^ cells. **c** Staining with anti-CD1a, anti-CD69 antibodies. **d** Staining with anti-CD4 and anti-CD69 antibodies. *Arrows* indicate CD69^+^ CD4^+^ cells. **e** A Western blot analysis revealed that the anti-survivin antibody reacted to all three variants. On the other hand, anti-survivin-ΔEx3 and anti-survivin-2B antibodies reacted with each respective variant only. **f** Immunohistochemistry (IHC) using anti-survivin-ΔEx3 and -2B antibodies (stained by red). *Right figures* show positive controls on the same slide

We first investigated the expressions of several IAP proteins in SSc and non-SSc skin lesions using IHC. The expression patterns of both XIAP and cIAP were similar (Fig. 1a-d), while only the survivin expression differed between SSc and non-SSc skin lesions (Fig. 1e-f). Survivin-overexpressing cells were detected in SSc dermis frequently. Subsequently, we performed the IHC analyses using anti-survivin antibodies on skin specimens obtained from 14 SSc patients and nine non-SSc patients (five cases of RA, one case of PM/DM and three cases of OA). Survivin-positive small cells were detected in the SSc dermal lesions. These cells were detected in the SSc dermis in 64.3 % of the cases (9/14), while they were rarely detected in the dermis from non-SSc patients (11.1 %, 1/9 cases) (*p* = 0.017) (Fig. 2a) (Table 1). Moreover, the rate of the coexistence of organ involvement in dermal survivin-positive SSc patients (62.5 %, 6/9) (three cases with interstitial pneumonia (IP), two cases with renal crisis (RC) and one case with coexisting RC and pulmonary artery hypertension (PAH)) was higher than that in dermal survivin-negative SSc patients (0 %, 0/5) (*p* = 0.028). Consequently, the dermal survivin may be a potential diagnostic marker of SSc with organ derangement.

**Table 1:** The characteristics of SSc patients who had skin specimens analyzed with IHC

Patient number	Age (decade)	Sex	Disease duration (year)	Disease subset	Survivin expression (dermis, IHC)	IP	RC	PAH	SSc-specific autoantibodies (positive,+; negative,-)		
									Scl-70	Centromere	RNAPIII
1	70s	Female	10	Limited	**+**	-	**+**	-	-	-	**+**
2	60s	Male	0.3	Limited	**+**	-	**+**	-	-	-	**+**
3	70s	Female	0.3	Diffuse	**+**	**+**	-	-	-	-	**+**
4	50s	Female	1	Limited	**+**	-	-	-	-	**+**	-
5	40s	Female	2	Limited	-	-	-	-	-	-	-
6	40s	Female	0.5	Limited	-	-	-	-	-	-	-
7	60s	Female	0.5	Limited	-	-	-	-	-	-	-
8	70s	Female	2	Limited	**+**	-	-	-	-	**+**	-
9	60s	Female	9	Limited	**+**	**+**	-	-	-	**+**	-
10	40s	Female	1	Limited	**+**	-	-	-	-	-	-
11	60s	Male	0.5	Limited	-	-	-	-	-	-	-
12	60s	Female	5	Limited	**+**	**+**	-	-	-	**+**	-
13	40s	Female	7	Diffuse	**+**	-	**+**	**+**	-	-	**+**
14	60s	Female	3	Limited	-	-	-	-	**+**	-	-

*SSc* systemic sclerosis, *IHC* immunohistochemistry, *IP* interstitial pneumonia, *RC* renal crisis, *PAH* pulmonary artery hypertension, *Scl-70* topoisomerase I, *RNAPIII* RNA polymerase III

### CD1a^+^ survivin^+^ cells in dermal lesions from SSc patients

Moreover, we determined the type of cells expressing survivin. The survivin-positive cells in the SSc dermis expressed CD1a antigen, one of the dendritic cell (DC) markers, using the multiple immunofluorescence method (Fig. 2b). Therefore, survivin in the SSc dermal lesions was expressed in CD1a^+^ DCs. Moreover, activated T lymphocytes (CD69^+^CD4^+^cells) existed around the CD1a^+^ cells (Fig. 2c-d).

### The survivin expression levels in PBMCs from SSc patients

Human dermal DCs mainly divide into two subsets: CD1a^+^ and CD14^+^ [18]. Conventional dermal DCs mainly develop from blood-derived DC precursors, in particular monocytes [19]. Therefore, we investigated the survivin expression levels of PBMCs and CD14^+^ cells from SSc patients and healthy controls (HCs). In these experiments, we collected PBMCs from SSc patients with IP and/or other organ involvement (Table 2), because many of the SSc patients with dermal survivin expression had organ derangement (Table 1).

**Table 2:** The characteristics of SSc patients who had PBMCs analyzed with RT-PCR and/or FACS analyses

Patient number	Age (decade)	Sex	Disease duration (year)	Disease subset	IP	RC	PAH	SSc-specific autoantibodies (positive,+; negative,-)			Anti-survivin antibody (serum, IgG)
								Scl-70	Centromere	RNAPIII	
1	70s	Female	19	Diffuse	**+**	-	**+**	**+**	-	**-**	**-**
2	80s	Female	4	Limited	**+**	-	-	-	-	**-**	**+**
3	50s	Female	2	Diffuse	**+**	-	**+**	-	-	**-**	**+**
4	50s	Female	22	Diffuse	**+**	-	-	**+**	**-**	-	**+**
5	50s	Male	22	Diffuse	**+**	**+**	**+**	-	-	**+**	**+**

*SSc* systemic sclerosis*, PBMCs* peripheral blood mononuclear cells, *IP* interstitial pneumonia, *RC* renal crisis, *PAH* pulmonary artery hypertension, *Scl-70* topoisomerase I, *RNAPIII* RNA polymerase III, *IgG* immunoglobulin G

**Fig. 3:**
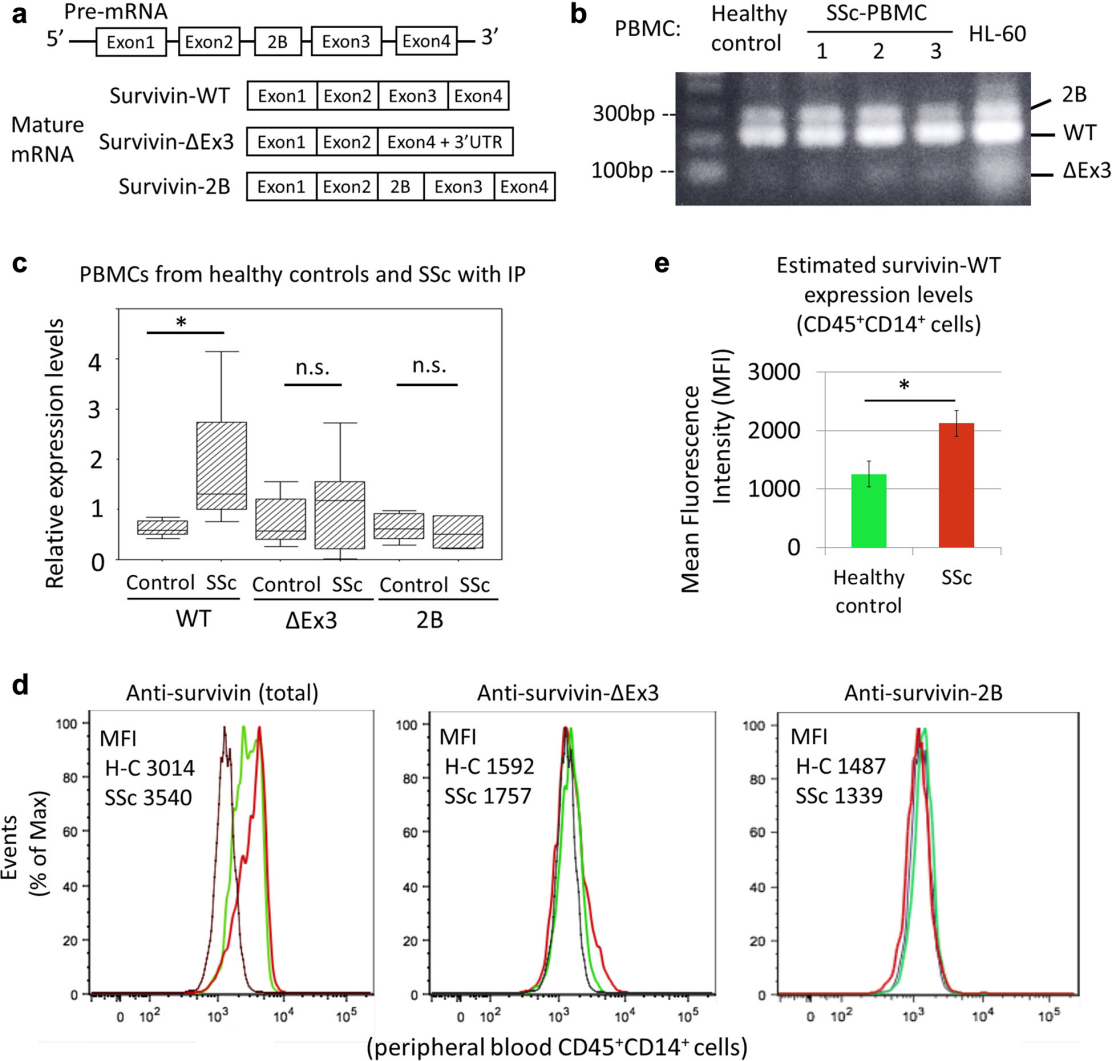
The expression levels of survivin splice variants in peripheral blood mononuclear cells (PBMCs) from systemic sclerosis (SSc) patients. **a** The scheme of survivin splice variants. *BIRC5* gene could generate several splice variants. **b** The reverse transcription polymerase chain reaction (RT-PCR) analysis of PBMCs from healthy controls (HCs) (n = 5) and SSc patients with interstitial pneumonia (IP) (n = 5). The figure shown is representative of the samples. **c** Relative expression levels of survivin (-WT, -ΔEx3 and -2B) in PBMCs from HCs (n = 5) and SSc patients (n = 5) using real-time PCR. ^*^Mann–Whitney *U* test, *p* < 0.05. **d** PBMCs were collected from HCs (n = 4) and SSc patients (n = 3), and fluorescence-activated cell sorting (FACS) analyses were performed for the measurement of intracellular survivin expression. The SSc patients were designated as numbers *1*, *3* and *5* in Table 2. Stained PBMCs were gated using the forward and side scatters and CD45^+^CD14^+^ cells were analyzed (*gray line*, normal immunoglobulin G (IgG) control; *green line*, HC; *red line*, SSc). Representative images and their mean fluorescence intensity (MFI) are shown. **e** Estimated survivin-wild type (WT) expression levels of peripheral CD45^+^CD14^+^ cells in HCs (n = 4) and SSc patients (n = 3). Mean ± SEM, ^*^ *t* test, *p* < 0.05

It was reported that the *BIRC5* gene could generate survivin splice variants, which result from alternative splicing (Fig. 3a) [20]. RT-PCR revealed that the expressions of survivin-WT, -ΔEx3 and -2B were detectable in PBMCs from SSc patients (n = 5) and HCs (n = 5) (Fig. 3b). Subsequently, we performed quantification of the survivin splicing variants using real-time PCR. As a result, the expression levels of only survivin-WT in PBMCs from SSc patients were higher than those from the controls (*p* = 0.016) (Fig. 3c). There were no significant differences in the expression levels of survivin-ΔEx3 or -2B. Moreover, we also measured the intracellular expression levels of survivin proteins in PBMCs using flow cytometry. As shown in Fig. 3d, flow cytometry using anti-survivin antibody, which can detect all survivin variants (Fig. 2e), revealed that the survivin expression levels in monocytes from SSc patients (CD45^+^CD14^+^ cells) were higher than those from HCs (Fig. 3d). However, the expression levels of survivin-ΔEx3 and -2B in CD45^+^CD14^+^ cells were similar between SSc patients and HCs. The estimated survivin-WT expression levels of CD45^+^CD14^+^ cells in SSc patients (n = 3) were higher than those in HCs (n = 4) (*p* = 0.043) (Fig. 3e).

These results did not contradict the IHC data for SSc dermal lesions, in which survivin-ΔEx3 or survivin-2B were not detected (Fig. 2f). Accordingly, these data suggest that survivin-WT is expressed in PBMCs, in particular CD14^+^ monocytes, and dermal CD1a^+^ DCs derived from PBMCs also express survivin-WT in SSc patients.

### Survivin-WT promotes cell proliferation in CD1a^+^ Mo-DCs

**Fig. 4:**
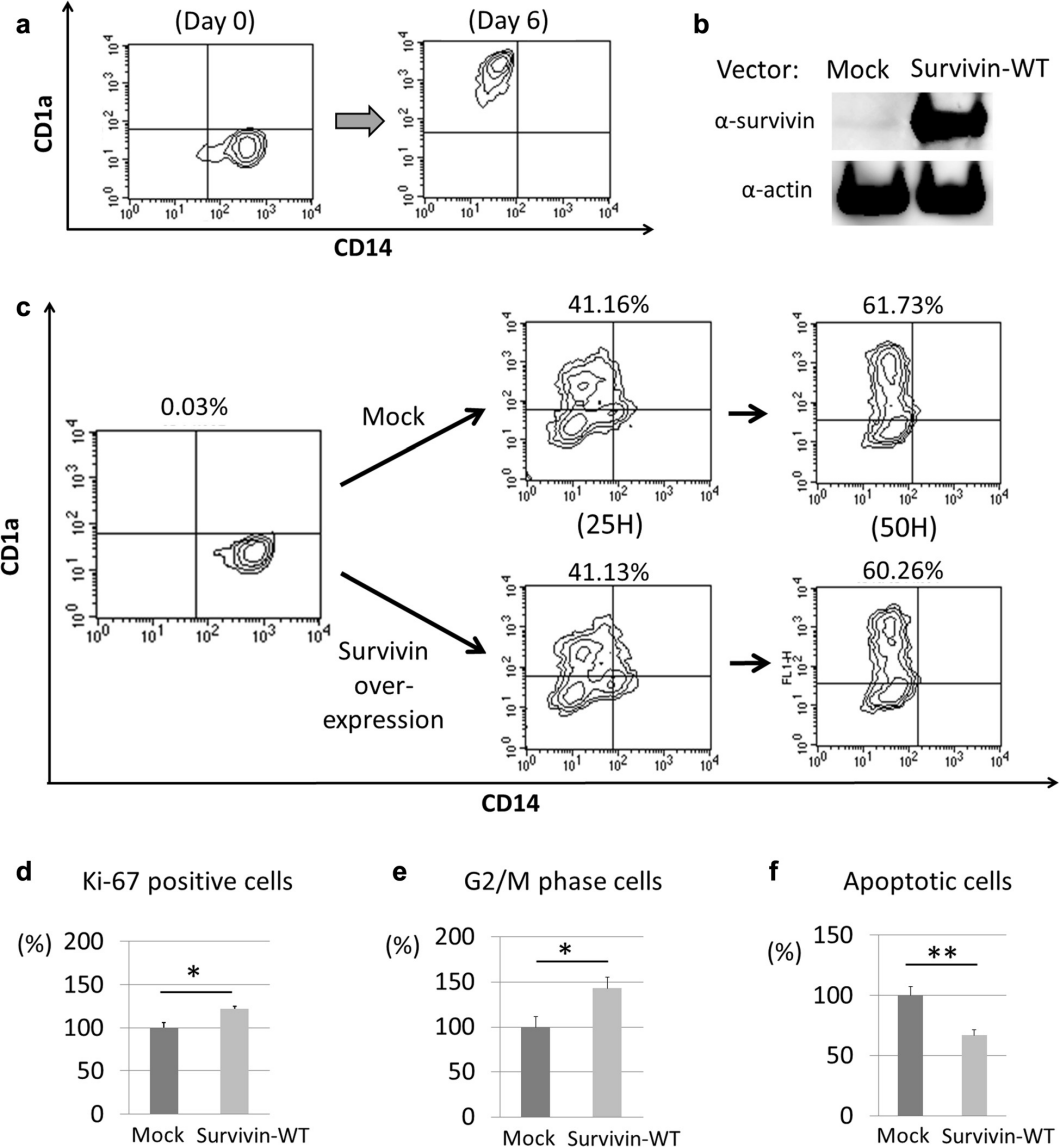
The role of survivin in CD1a^+^ monocyte-derived dendritic cells (Mo-DCs). **a** CD14^+^ monocytes change into CD1a^+^CD14^−^ DCs by culturing with granulocyte macrophage colony-stimulating factor (GM-CSF) and interleukin (IL)-4. **b** A Western blot analysis showed that BIRC5 mRNA expressed the survivin-WT protein in Mo-DCs. **c** Mo-DCs were transfected with survivin-wild type (WT) and mock at day 0, and were subsequently stimulated with GM-CSF and IL-4. At 25 and 50 hours, the samples were analyzed using fluorescence-activated cell sorting (FACS) analyses. The percentages in the figures indicate the ratio of CD1a^+^CD14^−^ cells. **d-f** Mo-DCs were transfected with survivin-WT and mock at day 6, and the following FACS analyses were performed at 24 hours. **d** Harvested Mo-DCs were stained with FVD520 and anti-Ki-67 antibodies. The positive rate was calculated as the number of Ki-67-positive cells among FVD520-negative (living) cells. Data are presented as the percentage of control cells. Mean ± SEM. n = 4 (both groups). ^*^ *t* test, *p* < 0.05. **e, f** Harvested Mo-DCs were stained with 7-amino-actinomycinD (7-AAD). The percentages of G2/M phase cells (**e**) and apoptotic cells (**f**) were calculated. Data are presented as the percentage of control cells. Mean ± SEM, n = 5 (both groups). *t* test, ^*^ *p* < 0.05, ^**^ *p* < 0.01

CD1a^+^ DCs can be generated from PBMCs by culturing in vitro [21] (Fig. 4a). We obtained Mo-DCs from healthy donors, established a transfection method (Fig. 4b), and investigated the function of overexpressed survivin. We selected survivin-WT among the splice variants, according to the results from PBMCs and IHC studies (as mentioned above). When Mo-DCs were transfected to overexpress survivin at day 0 (Fig. 4c), survivin overexpression did not affect the differentiation of DCs until 50 hours.

Using these systems, we investigated the role of survivin in CD1a^+^DCs. CD1a^+^ Mo-DCs were transfected to overexpress survivin at day 5. As a result, the Ki-67-positive rates, which reflect cell cycle progression, of survivin-overexpressing CD1a^+^ Mo-DCs were higher than those of controls (*p* = 0.019) (Fig. 4d). These cells were then stained with 7-AAD, which indicates cell cycling and DNA fragmentation. This analysis revealed that the rates of G2/M phase cells among the survivin-overexpressing DCs was higher than those among control cells (*p* = 0.034), and the rates of apoptotic cells among survivin-overexpressing DCs was lower than controls (*p* = 0.004). In conclusion, survivin-WT may be involved in CD1a^+^ Mo-DC proliferation through cell cycle activation and resistance to apoptosis. There were no significant differences in the expression levels of IL-6 and IL-12B between survivin-overexpressing DCs and controls (data not shown).

## Discussion

The pathological features of SSc skin lesions are a thin epidermis and thick dermis [22]; therefore, the dermal lesions of SSc may be noteworthy. On the other hand, Koike et al. reported that the levels of survivin proteins and autoantibodies against survivin were elevated in sera of SSc patients [9]. However, previous reports on the relationship among SSc, survivin and dermal DCs have been limited. In the present study, we detected the survivin expression in dermal lesions and PBMCs from SSc patients. The dermal survivin expression were observed especially in SSc patients with organ involvement. Therefore, we believe that survivin may be an attractive biomarker of SSc with organ derangement. For the detection of survivin expression in SSc patients, dermal IHC staining is a predominant method of choice. However, malignant tumor invasion, viral infections (e.g., herpes virus) or postoperative granulation tissue often express survivin in dermal lesions, which may lead to false positive results.

Survivin^+^ dermal cells also expressed CD1a, which is one of the DC markers. CD1a protein is a family of major histocompatibility complex (MHC) class I-like antigen-presenting molecules, which can combine with β-2-microglobulin and some apolar oil antigens and react to the CD1a-reactive αβT cell receptor [23]. CD1a-autoreactive T lymphocytes tend to home in on the skin and produce cytokines (for example, interferon (IFN)-γ, IL-22, etc.) [24]. Although CD1a^+^ DCs rarely exist in the normal dermis [25], many CD1a^+^ DCs existed in the SSc dermal lesions. Moreover, activated lymphocytes (CD69^+^CD4^+^) existed around these cells. According to our presented data, we speculate that CD1a^+^ survivin^+^ cells may induce an immunological reaction in the SSc dermal lesion.

A genome-wide association study (GWAS) showed that *IRF8* (interferon regulatory factor 8) gene was associated with SSc [26]. IRF8 is expressed at very high levels in mononuclear phagocytes and regulates granulocyte/macrophage differentiation and DC development in mouse models [27–30]. In humans, autosomal recessive IRF8 deficiency induces Mendelian susceptibility to mycobacterial disease (MSMD) [31]. In these patients, CD11c^+^ myeloid DCs or CD123^+^ plasmacytoid DCs could not be detected in the blood, and CD1a^+^ DCs were also not detected in the dermis. Therefore, some pathophysiological relationship may exist between DCs and SSc.

Conventional dermal DCs mainly develop from blood-derived DC precursors, in particular monocytes [19]. We speculate that PBMCs have a potential as DC precursors. We subsequently performed experiments to determine the expression levels of survivin in PBMCs from SSc patients using RT-PCR (mRNA expression) and FACS (protein expression). The *BIRC5* gene can generate several survivin variants, as noted above [20], and three variants (survivin-WT, -ΔEx3 and -2B) were dominantly expressed. However, survivin-WT-specific antibody could not be developed, because all amino acid sequences of survivin-WT were included in survivin-2B. As shown in Fig. 2e, the anti-survivin antibody used in our presented experiments reacts to all three variants. As an alternative method of survivin-WT protein analysis, we calculated the estimated survivin-WT expression levels using other parameters. The results of our FACS analysis demonstrated no difference in the expression levels of survivin-ΔEx3 or -2B, whereas survivin-WT was overexpressed in PBMCs (in particular CD14^+^ cells) from SSc patients compared with HCs. Moreover, the fact that neither survivin-ΔEx3 nor -2B was detected in SSc dermal lesions also supported the significance of survivin-WT expression. We believe that dermal survivin-WT^+^ DCs in SSc may be derived from peripheral blood monocytes.

Subsequently, we investigated the function of survivin-WT in CD1a^+^ DCs using in vitro cultured Mo-DCs. These experiments showed that survivin-WT could induce cell cycle activation and apoptosis inhibition in CD1a^+^ Mo-DCs. Therefore, we speculate that the role of survivin-WT in dermal CD1a^+^ DCs is cell proliferation through cell cycle activation and resistance to apoptosis.

## Conclusions

Our findings suggested that dermal survivin^+^ CD1a^+^ cell infiltration may be a potential biomarker of SSc skin lesions. PBMCs and monocytes from SSc patients also overexpressed survivin, therefore, dermal survivin^+^ DC may be derived from peripheral blood monocytes. Furthermore, survivin-WT could be involved in CD1a^+^ DC proliferation through cell cycle activation and resistance to apoptosis. Survivin may be an important molecule for the pathogenesis of SSc.

## Abbreviations

7-AAD: 7-amino-actinomycinD
AEC: 3-amino-9-ethylcarbazole
BIR: baculovirus IAP repeat
BIRC5: baculoviral IAP repeat containing 5
DAPI: 4′,6-diamidino-2-phenylindole, dihydrochloride
DC: dendritic cell
ELISA: enzyme-linked immunosorbent assay
FACS: fluorescence-activated cell sorting
GM-CSF: granulocyte macrophage colony-stimulating factor
GST: glutathione S-transferase
GWAS: genome-wide association study
HC: healthy control
HPRT: hypoxanthine-guanine phosphoribosyltransferase
HRP: horseradish peroxidase
IAP: inhibitor of apoptosis
IgG: immunoglobulin G
IHC: immunohistochemistry
IL: interleukin
INF: interferon
IP: interstitial pneumonia
IRF8: interferon regulatory factor 8
IVT: in vitro transcribed
MFI: mean fluorescence intensity
MHC: major histocompatibility complex
Mo-DC: monocyte-derived dendritic cell
mRNA: messenger RNA
MSMD: Mendelian susceptibility to mycobacterial disease
OA: osteoarthritis
PAH: pulmonary artery hypertension
PBMC: peripheral blood mononuclear cell
PM/DM: polymyositis/dermatomyositis
RA: rheumatoid arthritis
RC: renal crisis
RNAPIII: RNA polymerase III
RT-PCR: reverse transcription polymerase chain reaction
Scl-70: topoisomerase I
SSc: systemic sclerosis
TBS: Tris-buffered saline
WT: wild type
